# Eight Surgical Interventions for Lumbar Disc Herniation: A Network Meta-Analysis on Complications

**DOI:** 10.3389/fsurg.2021.679142

**Published:** 2021-07-20

**Authors:** Fei-Long Wei, Tian Li, Quan-You Gao, Yi Yang, Hao-Ran Gao, Ji-Xian Qian, Cheng-Pei Zhou

**Affiliations:** ^1^Department of Orthopedics, Tangdu Hospital, Fourth Military Medical University, Xi'an, China; ^2^School of Basic Medicine, Fourth Military Medical University, Xi'an, China; ^3^Department of Pain Treatment, Tangdu Hospital, Fourth Military Medical University, Xi'an, China

**Keywords:** lumbar disc herniation, minimally invasive surgery, network meta-analysis, reoperation, complication

## Abstract

**Objective:** Therapeutic options for lumbar disc surgery (LDH) have been rapidly evolved worldwide. Conventional pair meta-analysis has shown inconsistent results of the safety of different surgical interventions for LDH. A network pooling evaluation of randomized controlled trials (RCT) was conducted to compare eight surgical interventions on complications for patients with LDH.

**Methods:** PubMed, Embase, and the Cochrane Central Register of Controlled Trials (CENTRAL) were searched for RCT from inception to June 2020, with registration in PROSPERO (CRD42020176821). This study is conducted in accordance with Cochrane guidelines. Primary outcomes include intraoperative, post-operative, and overall complications, reoperation, operation time, and blood loss.

**Results:** A total of 27 RCT with 2,948 participants and eight interventions, including automated percutaneous lumbar discectomy (APLD), chemonucleolysis (CN), microdiscectomy (MD), micro-endoscopic discectomy (MED), open discectomy (OD), percutaneous endoscopic lumbar discectomy (PELD), percutaneous laser disc decompression (PLDD), and tubular discectomy (TD) were enrolled. The pooling results suggested that PELD and PLDD are with lower intraoperative and post-operative complication rates, respectively. TD, PELD, PLDD, and MED were the safest procedures for LDH according to complications, reoperation, operation time, and blood loss.

**Conclusion:** The results of this study provided evidence that PELD and PLDD were with lower intraoperative and post-operative complication rates, respectively. TD, PELD, PLDD, and MED were the safest procedures for LDH according to complications, reoperation, operation time, and blood loss.

**Systematic Review Registration:** PROSPERO, identifier CRD42020176821.

## Introduction

Lumbar disc herniation (LDH) is highly associated with inflammation in the context of low back pain (1). It is a common disease in spine surgery and a primary cause of sciatica, which affects 1–2% of the general population in the USA annually (2, 3). Approximately, 5% of men and 2.5% of women will experience sciatica at some point in their lives (4). Many cases of acute sciatica can be treated conservatively with satisfactory results (5). Conservative treatment as a first-line treatment can benefit most patients with LDH (6–8). However, surgical treatment is considered a more effective way for rapid pain relief and nerve decompression (5, 9). Surgical methods including traditional discectomy and minimally invasive techniques have become more popular in recent years (10, 11).

Open discectomy (OD) has been considered the standard surgical treatment for lumbar disc herniation since 1929 (12). Currently, microdiscectomy (MD) displayed by microscope was introduced in 1976 and is identified as the gold standard procedure for treating LDH with better visualization (13). Automated percutaneous lumbar discectomy (APLD) was reported in 1985 using a new aspiration probe (14). Micro-endoscopic discectomy (MED) technology, introduced by Foley in 1997, is displayed by microendoscope performing by a transmuscular approach and has less damage to soft tissues and muscles than MD (15). With the advancement of spine endoscopy, minimally invasive surgery for symptomatic LDH (16) has been further developed to allow patients to suffer smaller surgical trauma and thereby recover faster, such as percutaneous laser disc decompression (PLDD) with laser energy delivered to the nucleus pulposus by means of fiber (17), and percutaneous endoscopic lumbar discectomy (PELD) including introduced percutaneous endoscopic interlaminar discectomy and percutaneous endoscopic transforaminal discectomy (18). Chemonucleolysis (CN), introduced in 1964, is the injection of proteolytic enzymes into the disc cavity to dissolve displaced disc material (19). The tubular discectomy (TD) system is a muscular split tubular approach that was proposed in 1997 (20). It allows surgeons to work with two hands through a small-diameter, operating table-mounted tubular retractor.

A novel surgical approach has brought some complications while benefiting patients with LDH. However, the current studies have not yet compared all interventions and analyzed their advantages and disadvantages (12, 21, 22). We use a network meta-analysis (NMA) of multiple treatments to provide a clinically useful conclusion based on the results of NMA, which can be used to guide treatment decisions.

## Materials and Methods

### Search Strategy

This study was registered in PROSPERO (CRD42020176821). We searched the PubMed, Embase, and the Cochrane Central Register of Controlled Trials (CENTRAL) based on the preferred reporting information for systematic reviews and meta-analyses (PRISMA) guidelines to identify all relevant studies published until June 2020 (23–25). Keywords and mesh terms for the searching strategy include “lumbar disc herniation,” “open discectomy,” “intervertebral discectomy,” “microdiscectomy,” “minimally invasive surgery,” “percutaneous disc Resection,” and “laser.” Articles written only in English were included in this study.

### Enrolled Criteria

Please refer to Supplementary Table 1.

### Study Selection

The two authors of the review team (Wei and Li) independently reviewed all titles and abstracts found in the database during the initial online search and excluded papers that were not relevant to the subject. The full-text articles of all relevant abstracts were further reviewed. In the research selection process, disagreements between reviewers can be resolved by third-party reviewers (Zhu).

### Data Extraction

A standard “characteristics of included studies” table from the Cochrane Handbook (26), was piloted in parallel with the development of the search strategy and modified to match the needs of this review. Data extraction was performed independently by two reviewers (Wei and Du). A third reviewer (Gao) checked the accuracy of the extracted data. If the data needed was missing from the paper, the study authors who participated in the review was contacted. The extracted data included study design, sample size, inclusion and exclusion criteria, study time, mean follow-up time, number of participants, age, gender, interventions, and outcomes (operation time, blood loss, number of complications, and reoperations).

### Risk of Bias Within Individual Studies

This study used 13 criteria recommended in the Cochrane Back and Neck Group guidelines (27) to assess the risk of bias (Supplementary Table 2).

### Summary Measures and Synthesis of Results

Pairwise meta-analyses for studies that directly compared different interventions were imported by RevMan 5.3 (Nordic Cochrane Center, Copenhagen, Denmark). NMA plots depicting were completed with statistical analysis software STATA 14.0 (StataCorp LLC, TX, USA). The NMA was performed by WinBUGS 1.4.3 (MRC Biostatistics Unit, Cambridge, UK) by a random effects model (REM) (28). Results obtained using the Markov Chain Monte Carlo (MCMC) method are reported as the median of the posterior distribution with 95% CI. Non-informative prior distributions and overdispersed initial values (with a scaling of five) were used in models in two chains to fit the model (29, 30), yielding 100,000 iterations (including 50,000 tuning iterations) and a thinning interval of 10 for each chain. Treatment inconsistency evaluation is an important aspect of NMA. It judges whether the treatment effect is consistent through direct evidence and indirect evidence. The results of node-splitting are used to evaluate the consistency of the direct and indirect comparisons; *P* < 0.05 indicated significant inconsistency. If there is significant heterogeneity, a random effects model was used. Otherwise, we use a fixed effects model.

### Evaluating the Quality of Evidence

Two reviewers (Wei and Zhou) independently assessed the quality of the evidence for each study by using the Cochrane Collaboration tool to assess the risk of bias (31). The quality of evidence was reported using the GRADE criteria (32, 33).

## Results

### Literature Search and Network Structure

**Figure 1:**
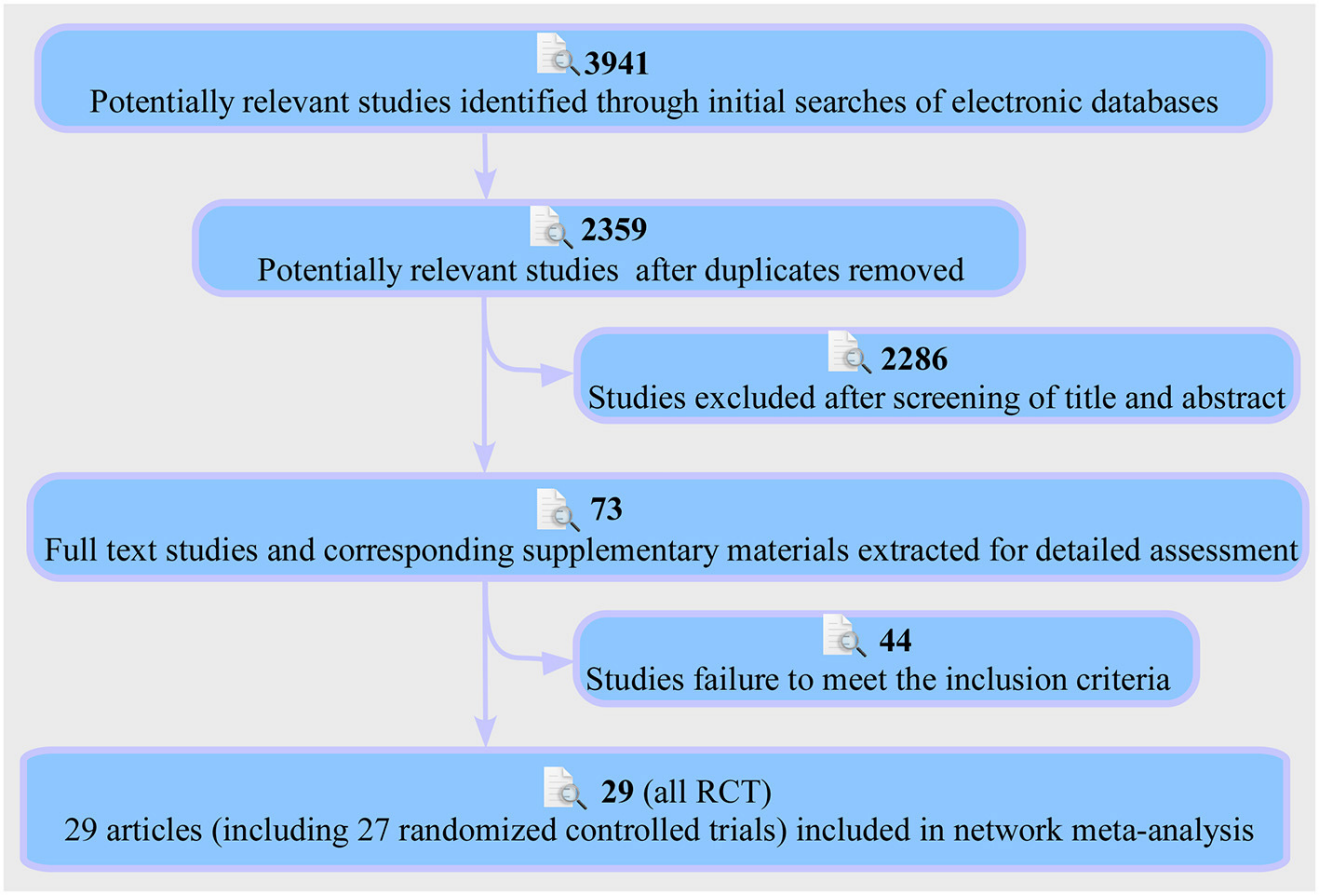
Flowchart of study selection and design.

**Figure 2:**
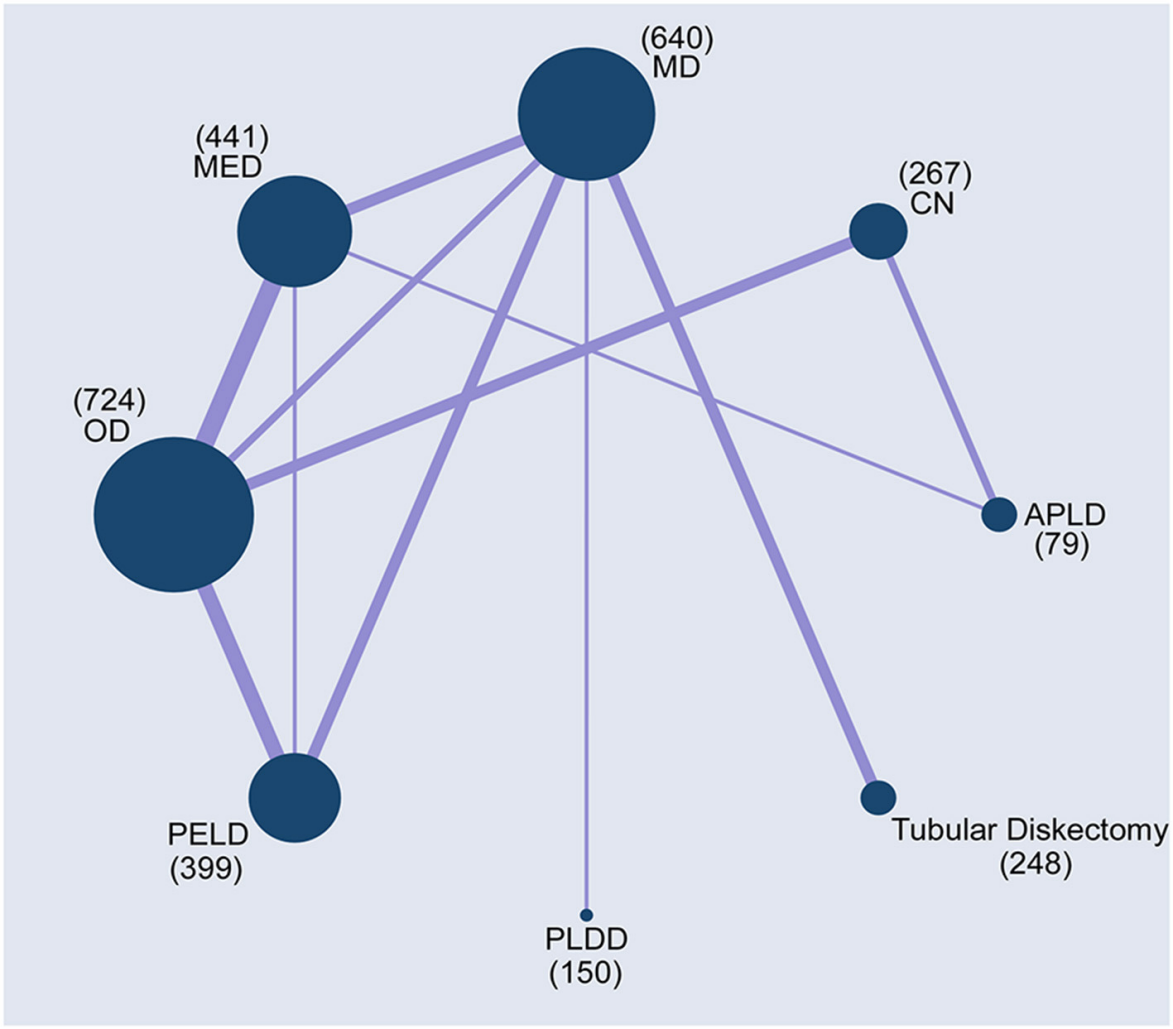
Network plots of comparisons based on network meta-analysis (NMA). Each circular node represented a type of treatment. The circle size is proportional to the total number of patients. The width of lines was proportional to the number of studies performing head-to-head comparisons in the same study.

The PRISMA flow chart of the selection process for the study was shown in Figure 1. This retrieved 3,941 results, and after filtering by title, 1,582 irrelevant papers or duplicates were deleted (Wei). The title and abstract of the remaining 2,359 studies were then screened, and the remaining 73 papers were assessed for full text (Wei, Yang). Finally, 29 published articles were eligible for inclusion in the study to perform a multi-therapy meta-analysis (Figure 2) (20, 34–61). The baseline characteristics of the 27 studies (27 RCT) with 2,948 participants are shown in Supplementary Table 3 (20, 34–59).

### Risk of Bias in Included Studies

The risk of selection bias for each study was described according to the Cochrane Back and Neck Group guidelines (27). Supplementary Figures 1, 2 showed a summary of the risk of bias assessment. Six studies were evaluated as high risk of selection bias (35, 41, 46, 47, 52, 53). Regarding the blinding method, 11 studies were considered as high risk (20, 35, 50, 51, 58, 59). No study had been evaluated as high risk of reporting bias. One study was assessed as having a high risk of outcome detection bias (56). In addition, no studies had been evaluated as high-risk of selective reports and other potential biases.

### Complication

#### Complication Based NMA

**Figure 3:**
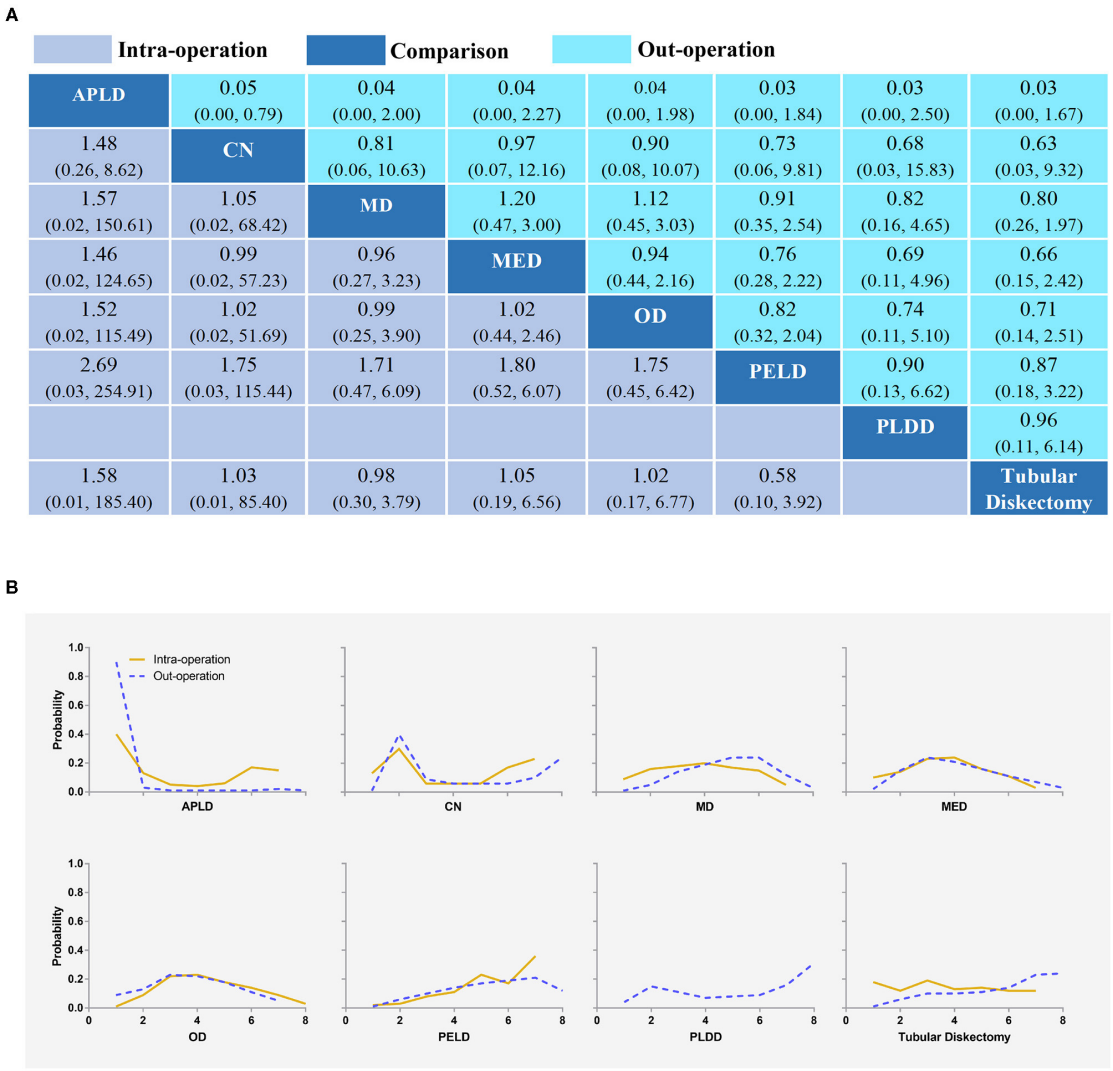
Intra-complication and out-complication **(A)** and the rank possibility of intra-complication and out-complication **(B)** based NMA in the consistency model. Each cell of the profile contained the pooled odds ratio (OR) and 95% credibility intervals for disability change; significant results were in bold. APLD, Automated percutaneous lumbar discectomy; CN, Chemonucleolysis; MD, Microdiscectomy; MED, Micro-endoscopic discectomy; OD, Open discectomy; PELD, Percutaneous endoscopic lumbar discectomy; PLDD, Percutaneous laser disc decompression.

**Figure 4:**
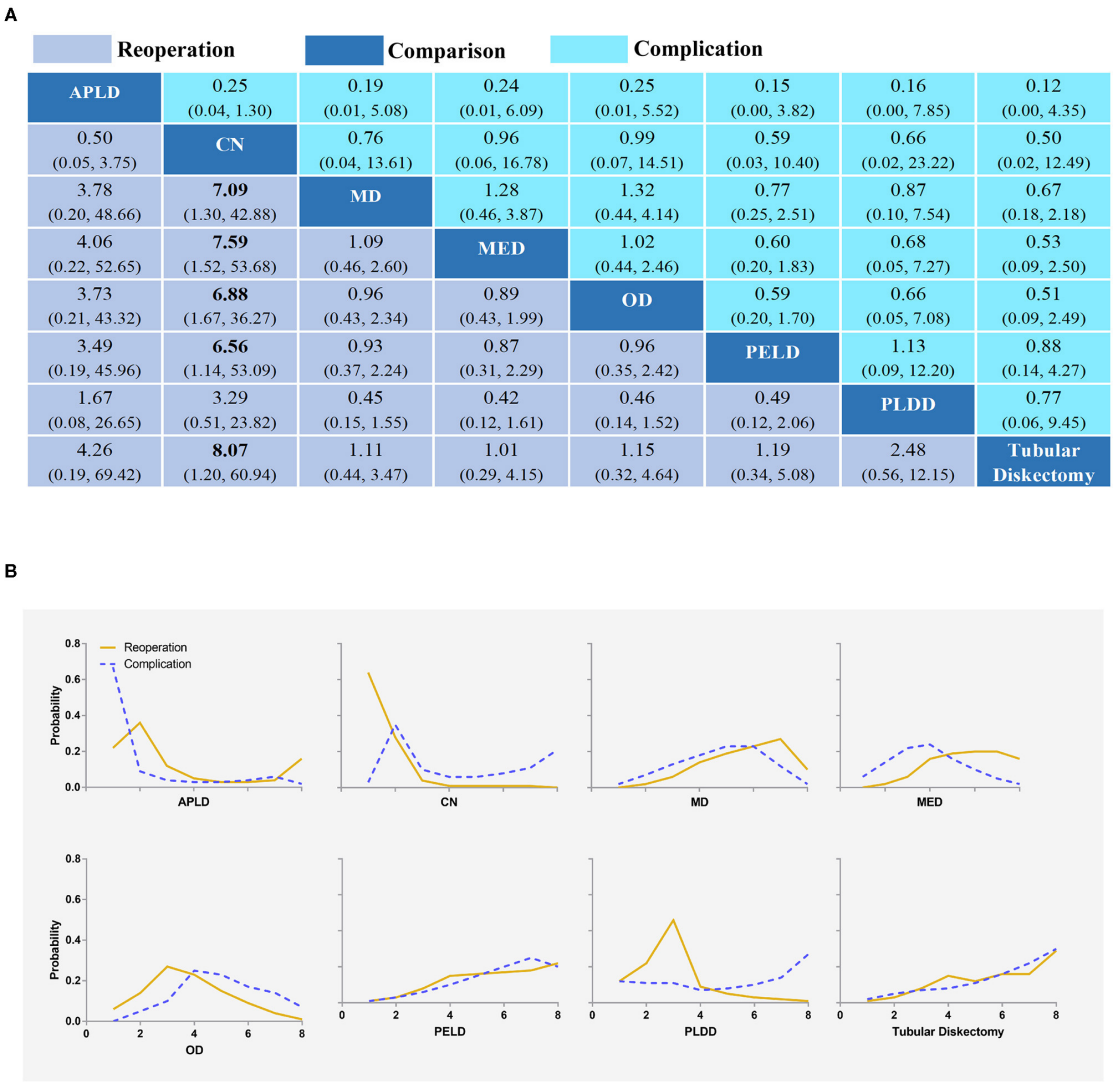
Overall complication and reoperation **(A)** and the rank possibility of overall complication and reoperation **(B)** based NMA in the consistency model. Each cell of the profile contained the pooled OR and 95% credibility intervals for disability change; significant results were in bold. APLD, Automated percutaneous lumbar discectomy; CN, Chemonucleolysis; MD, Microdiscectomy; MED, Micro-endoscopic discectomy; OD, Open discectomy; PELD, Percutaneous endoscopic lumbar discectomy; PLDD, Percutaneous laser disc decompression.

About 24 studies reported complications for statistical analysis (20, 34, 36, 37, 39–50, 52–59). Compared with other interventions, whether it was intraoperative, post-operative, or overall complications, PELD had a lower incidence of complications. However, the differences were not statistically significant (Figures 3A, 4A). The results obtained by the consistency model were in accordance with the results obtained by the inconsistency model, and there was no obvious inconsistency in the node split analysis (all *P* > 0.05, Supplementary Figure 3 and Supplementary Table 4).

#### The Rank Possibility of Complication Based NMA Inconsistency Model

The distribution of probabilities about the complication rate of each intervention being ranked at each of the possible eight positions is shown in Figures 3B, 4B. PELD was with the lowest intraoperative complication rate (Figure 3B). The cumulative probabilities of being among the lowest intraoperative complication rates were: PELD (36%), CN (23%), APLD (15%), TD (14%), MD (5%), OD (5%), and MED (3%). PLDD was with the lowest post-operative complication rate (Figure 3B). The cumulative probabilities of being among the lowest post-operative complication rates were: PLDD (31%), TD (24%), CN (24%), PELD (12%), MED (3%), MD (3%), OD (3%), and APLD (15%). In addition, TD, PLDD, CN, and PELD were with the lowest overall complication rates (Figure 4B). The cumulative probabilities of being among the lowest overall complication rates were: TD (29%), PLDD (26%), CN (20%), PELD (18%), MED (2%), MD (2%), APLD (1%), and OD (1%) and the probabilities were detailed in the Supplementary Table 5.

### Reoperation

#### Reoperation Based NMA

All 27 studies reported reoperations for statistical analysis (20, 34–59). TD had a lower incidence of reoperation. However, except that CN had higher reoperation rates than MD, MED, OD, PLED, and TD with statistical significance, there were no statistically significant differences in other comparisons (Figure 4A). The results obtained by the consistency model were in accordance with the results obtained by the inconsistency model, and there was no obvious inconsistency in the node split analysis (all *P* > 0.05, Supplementary Figure 4 and Supplementary Table 6).

#### The Rank Possibility of Reoperation Based NMA Inconsistency Model

The distribution of probabilities about reoperation rates of each intervention being ranked at each of the possible eight positions is shown in Figure 4B. TD was with the lowest reoperation rate (Figure 4B). The cumulative probabilities of being among the lowest reoperation rates were: TD (29%), MED (21%), PELD (15%), OD (11%), APLD (10%), MD (9%), PLDD (2%), and CN (0%). The probabilities were detailed in the Supplementary Table 7.

### Blood Loss

#### Blood Loss Based NMA

**Figure 5:**
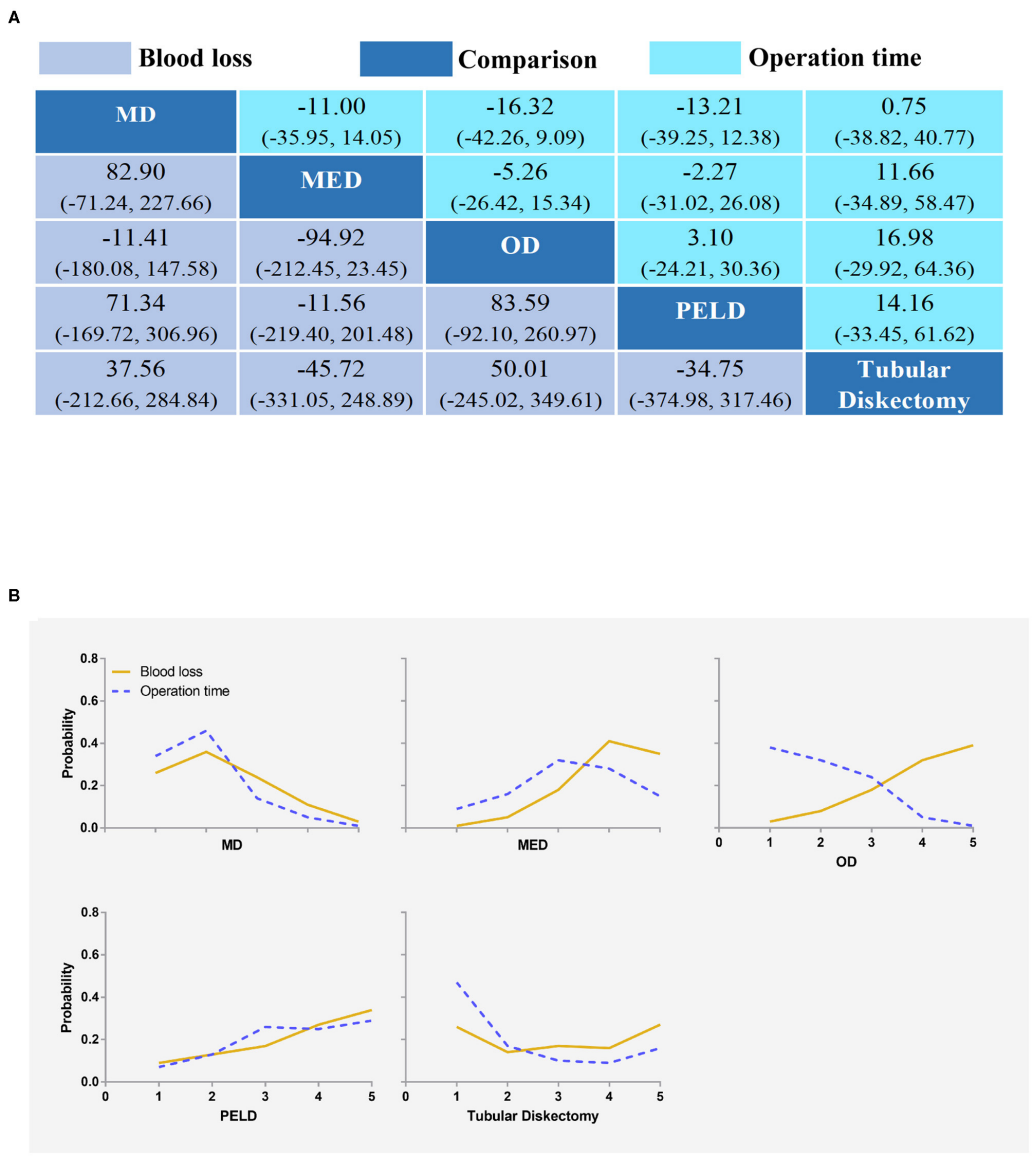
Blood loss and operation time **(A)** and the rank possibility of blood loss and operation time **(B)** based on NMA in the consistency model. Each cell of the profile contained the pooled mean difference and 95% credibility intervals for disability change; significant results are in bold. MD, Microdiscectomy; MED, Micro-endoscopic discectomy; OD, Open discectomy; PELD, Percutaneous endoscopic lumbar discectomy.

Ten studies reported blood loss for statistical analysis (34, 36, 43, 46, 47, 49, 54–57). MED had the least amount of blood loss. However, there was no statistically significant difference between any two interventions (Figure 5A). The results obtained by the consistency model were in accordance with the results obtained by the inconsistency model, and there was no obvious inconsistency in the node split analysis (all *P* > 0.05, Supplementary Figure 5 and Supplementary Table 8).

#### The Rank Possibility of Blood Loss Based NMA Inconsistency Model

The distribution of probabilities about blood loss of each intervention being ranked at each of the possible five positions is shown in Figure 5B. The cumulative probabilities of being among the least blood loss were: MED (35%), PELD (34%), TD (27%), MD (3%), and OD (1%). The probabilities were detailed in the Supplementary Table 9.

### Operation Time

#### Operation Time-Based NMA

Seventeen studies reported operation time for statistical analysis (20, 34, 36, 39, 42, 44, 46, 47, 49, 52–59). OD took the shortest time. However, there was no statistically significant difference between any two interventions (Figure 5A). The results obtained by the consistency model were in accordance with the results obtained by the inconsistency model, and there was no obvious inconsistency in the node split analysis (all *P* > 0.05, Supplementary Figure 5 and Supplementary Table 10).

#### The Rank Possibility of Operation Time-Based NMA Inconsistency Model

The distribution of probabilities about the operation time of each intervention being ranked at each of the possible five positions is shown in Figure 5B. The cumulative probabilities of being among the shortest operation time were: OD (39%), PELD (29%), TD (16%), MED (15%), and MD (1%). The probabilities were detailed in the Supplementary Table 11.

## Discussion

This NMA provided a hierarchical ranking of intraoperative, post-operative, and overall complications, reoperations, blood loss, and operation time for eight different interventions for LDH patients. In recent years, minimally invasive technologies have developed rapidly, including PELD, TD, MED, and MD. A total of 27 clinical RCT were included in this study. This study confirmed that they were all safe surgeries and had lower complication rates than traditional open surgery.

Compared with other interventions, whether it was intraoperative, post-operative, or total complications, APLD was with a higher incidence of complications. PELD was with the lowest intro-complication rate, which was consistent with prior studies (12, 62). PLDD was with the lowest out-complication rates. TD has the lowest complication rates, which was somewhat different from previous meta-analyses (21, 22). The previous studies believed that the overall complication rate was the lowest in PELD, and this study still had a low but not lowest complication rate in PELD. The reason for this result might be that all interventions were included reducing the incidence of bias. In addition, TD, PELD, and MED were good interventions for LDH. Their biggest advantage is that they do less damage to the spinal muscle and soft tissue, and better visualization, which makes the incidence of complications lower.

This study showed that the reoperation rate of CN was higher than MED and OD, which was statistically significant. And the result was consistent with the prior study (21). TD with the lowest reoperation rate was inconsistent with prior studies (12). This difference was due to the reason that previous studies were traditional paired meta-analyses and the number of studies included was small.

Blood loss and operation time are important indicators for evaluating surgical risks. MED surgery had the least blood loss, which was inconsistent with prior studies (63). Previous studies suggested that PELD had less blood loss than MED (63). This result might be because previous studies included many non-RCT and the quality of the included studies was not high. OD took the shortest operation time, but the difference was not statistically significant. However, previous studies have suggested that OD took a longer time than PELD (18). The reason for this result might be that this meta-analysis included some dated studies when the technology was not yet mature. With the advancement of PELD technology, the operation time is gradually shortened.

### Limitation

Although this study was somewhat different from previous studies, in general, the main results in this NMA were consistent with most previous reports. Previous studies without all interventions may be an important reason for this difference. Although the NMA incorporates all intervention methods and the results were relatively comprehensive, this study still had certain limitations. First, the size of the direct comparison and the sample size of many studies was small, which increases the instability of the statistical results. Second, because the prognostic indicators were reported at different time points, there was great heterogeneity. In addition, different standards of complications reported by different researchers may cause heterogeneity. So, there is an urgent need to further study and formulate a standard complication evaluation scheme for LDH discectomy. Whether PELD is better than TD and PLDD still needs to be confirmed in head-to-head randomized trials.

In conclusion, the results of this NMA provided evidence that PELD and PLDD were the safest procedures for LDH with minimal intraoperative and post-operative complications, respectively. TD, PELD, PLDD, and MED were the safest procedures for LDH according to complications, reoperation, operation time, and blood loss. The importance of this study can be used to guide treatment decisions.

## Data Availability Statement

The raw data supporting the conclusions of this article will be made available by the authors, without undue reservation.

## Author Contributions

All authors contributed substantially to the conception and design of the work, acquisition and interpretation of data, and the drafted work. F-LW, J-XQ, C-PZ, TL, and Q-YG contributed to the revised the work critically. All authors have approved the final version to be published and have agreed to be accountable for all aspects of the work.

## Conflict of Interest

The authors declare that the research was conducted in the absence of any commercial or financial relationships that could be construed as a potential conflict of interest.
