# Supplementary material for: Eight Surgical Interventions for Lumbar Disc Herniation: A Network Meta-Analysis on Complications
Source: Front Surg. 2021 Jul 20;8:679142. doi: 10.3389/fsurg.2021.679142 (PMC8329383; doi:10.3389/fsurg.2021.679142)
Supplement: Supplementary file 1 [file Table_1.docx]

**Table S1. Inclusion/exclusion criteria of literature**

| **PICOS** | **Inclusion** | **Exclusion** |
| --- | --- | --- |
| P | Adults (≥18 y) with LDH who have indications for surgical intervention, irrespective of age, gender, and race | 1) Children, pregnant women;  2) Patients with unexplained low back pain. |
| I | 1) Therapy that included 2 of the 8 interventions;  2) No limit on sample size. | Therapy: Except those eight treatment methods were excluded. |
| C | Therapy that included 2 of the 8 interventions. | Therapy: Except those eight treatment methods were excluded. |
| O | 1) Primary outcomes including intra-operation, out-operation, overall complication, and reoperation.  2) Secondary outcome included blood loss and operation time. | Not available. |
| S | RCT irrespective of blinding or arm. | 1) Articles without peer-reviewed or unpublished;  2) Studies that were repeatedly published or had qualitative outcomes;  3) Quasi-experimental studies, crossover, and observational studies. |
