# Supplementary material for: Eight Surgical Interventions for Lumbar Disc Herniation: A Network Meta-Analysis on Complications
Source: Front Surg. 2021 Jul 20;8:679142. doi: 10.3389/fsurg.2021.679142 (PMC8329383; doi:10.3389/fsurg.2021.679142)
Supplement: Supplementary file 2 [file Table_2.docx]

**Table S2. Risk of bias table.**

| 1 | Random sequence generation (selection bias) |
| --- | --- |
| 2 | Allocation concealment (selection bias) |
| 3 | Blinding of participants (performance bias) |
| 4 | Blinding of personnel/care provider (performance bias) |
| 5 | Blinding of outcome assessment (detection bias) |
| 6 | Incomplete outcome data (attrition bias) |
| 7 | Selective reporting (reporting bias) |
| 8 | Group similarity at baseline (selection bias) |
| 9 | Intention-to-treatment-analysis |
| 10 | Compliance (performance bias) |
| 11 | Co-interventions (performance bias) |
| 12 | Turning of outcome assessment (detection bias) |
| 13 | Other bias |
