# Supplementary material for: Eight Surgical Interventions for Lumbar Disc Herniation: A Network Meta-Analysis on Complications
Source: Front Surg. 2021 Jul 20;8:679142. doi: 10.3389/fsurg.2021.679142 (PMC8329383; doi:10.3389/fsurg.2021.679142)
Supplement: Supplementary file 3 [file Table_3.docx]

| **Table S3. Basic data** | | | | | | | | | | | | |
| --- | --- | --- | --- | --- | --- | --- | --- | --- | --- | --- | --- | --- |
| **Number** | **Studies** | **Study Country** | **Surgery Type** | **Numbe of Participants** | **Age**  **(Mean±SD)** | **Gender (M/F)** | **Follow-up**  **(m)** | **Operation time (min)** | **Blood loss (ml)** | **No. of** **Complications** | | **No. of**  **Reoperations** |
|  |  |  |  |  |  |  |  |  |  | **intra-operation** | **post-operation** |  |
| 1 | Bhavuk Garg  2011 | RCT  India | MED | 55 | 37 ±8 | 39/19 | 12 | 84 ±36 | 41 ±12 | 5 | 7 | 1 |
|  |  |  | OD | 57 | 38 ±6 | 44/13 | 12 | 56 ±33 | 306 ±120 | 5 | 10 | 0 |
| 2 | Crawshaw,  1984 | RCT  England | CN | 89 | 40 ±10 | NA | 12 | NA | NA | NA | NA | 19 |
|  |  |  | OD | 27 | 42 ± 9 | NA | 12 | NA | NA | NA | NA | 1 |
| 3 | Thomé Claudius  2005 | RCT  Germany | MD | 42 | 40 ±10 | 23/19 | 12 | 38.2±10.3 | 8.2 ±61.6 | NA | 1 | 4 |
|  |  |  | OD | 42 | 42 ± 9 | 24/18 | 12 | 32.6±13.8 | 67.0 ±85.4 | NA | 0 | 2 |
| 4 | Douglas Wardlaw  2013 | RCT  USA | CN | 48 | NA | 27/21 | 12 | NA | NA | 0 | 0 | 1 |
|  |  |  | OD | 52 | NA | 33/19 | 12 | NA | NA | 1 | 0 | 0 |
| 5 | Frank U. Hermantin  1999 | RCT  New Orleans | OD | 30 | 40 (18-67) | 17/17 | 31 | NA | NA | 1 | 1 | 1 |
|  |  |  | PELD | 30 | 39 (15-66) | 22/8 | 32 | NA | NA | 0 | 0 | 0 |
| 6 | Mayer H. M.  1993 | RCT  Germany | PELD | 20 | 39.8 ±10.4 | 12/8 | 24 | 40.7 ±11.3 | NA | NA | NA | 3 |
|  |  |  | MD | 20 | 42.7 ±10 | 14/6 | 24 | 58.2 ±15.2 | NA | NA | NA | 0 |
| 7 | Krugluger  2000 | RCT  Austria | CN | 12 | 37 (24-54) | 8/4 | 24 | NA | NA | 0 | 0 | 1 |
|  |  |  | APLD | 10 | 42 (26-60) | 8/2 | 24 | NA | NA | 1 | 5 | 1 |
| 8 | Jörg Franke  2009 | RCT  Germany | MD | 48 | 44 (21-72) | 30/20 | 12 | NA | NA | 3 | 5 | 5 |
|  |  |  | TD | 52 |  | 30/21 | 12 | NA | NA | 2 | 2 | 2 |
| 9 | J. N. Alaistair Gibson  2017 | RCT  UK | PELD | 70 | 42 ±9 | 30/40 | 24 | 61 ±16 | NA | 2 | 4 | 5 |
|  |  |  | MD | 70 | 39 ±9 | 40/30 | 24 | 65 ±36 | NA | 0 | 1 | 2 |
| 10 | Lei Pan  2014 | RCT  China | PELD | 10 | NA | NA | 6 | NA | 8.35 ±2.99 | NA | 1 | 0 |
|  |  |  | OD | 10 | NA | NA | 6 | NA | 99.00 ± 22.33 | NA | 0 | 0 |
| 11 | Marco Teli  2010 | RCT  Italy | MED | 70 | 39 ± 12 | 45/25 | 26±2 | 56 ± 12 | NA | 8 | 11 | 8 |
|  |  |  | MD | 72 | 40 ± 12 | 48/24 | 26±3 | 43 ±8 | NA | 2 | 8 | 4 |
|  |  |  | OD | 70 | 39 ± 12 | 46/24 | 26±2 | 36 ± 10 | NA | 2 | 5 | 3 |
| 12 | M. Revel  1993 | RCT  Germany | CN | 72 | 40 ± 10 | 47/25 | 24 | NA | NA | 5 | NA | 3 |
|  |  |  | APLD | 69 | 37± 8 | 47/22 | 24 | NA | NA | 7 | NA | 1 |
| 13 | Mark P. Arts  2011 | RCT  the Netherlands | TD | 166 | 41.6 ± 9.8 | 84/82 | 24 | 47 ± 22 | NA | 20 | 19 | 23 |
|  |  |  | MD | 159 | 41.3 ± 11.7 | 88/71 | 24 | 36 ± 16 | NA | 13 | 14 | 14 |
| 14 | Mohamed Hussein  2014 | RCT  Egypt | MED | 95 | 30.2 | 58/42 | 104.2 | 98.8 ±26.9 | 41.6 ±13.1 | 5 | 13 | 6 |
|  |  |  | OD | 90 | 31.5 | 54/46 | 101.3 | 97.27 ±13.5 | 124.22 ±24.5 | 6 | 8 | 9 |
| 15 | Mohamed Hussein  2016 | RCT  Egypt | MED | 37 | 30.5 | 20/17 | 25.5 | 91 ±8.5 | 35.4 ±10.2 | 1 | 3 | 3 |
|  |  |  | MD | 36 | 31.9 | 21/15 | 26.2 | 160.5 ±12.4 | 126.34 ±25.8 | 2 | 7 | 7 |
| 16 | Muralikuttan  1992 | RCT  UK | CN | 46 | 36 | 27/19 | 12 | NA | NA | NA | 1 | 9 |
|  |  |  | OD | 46 | 39 | 28/18 | 12 | NA | NA | NA | 0 | 1 |
| 17 | Orlando Righesso  2007 | RCT  Brazil | MD | 19 | 46.0 ±12.4 | 13/6 | 24 | 63.7 ± 15.5 | 40 ±109.8 | 0 | 1 | 1 |
|  |  |  | MED | 21 | 42.0 ±10.7 | 10/11 | 24 | 82.6 ±21.9 | 50 ±172.5 | 1 | 2 | 1 |
| 18 | Patrick A Brouwer  2017 | RCT  the Netherlands | PLDD | 55 | 43,2 ± 11,8 | 35/19 | 24 | NA | NA | NA | 6 | 29 |
|  |  |  | MD | 57 | 43,7 ± 9,7 | 42/24 | 24 | NA | NA | NA | 7 | 12 |
| 19 | Saeid Abrishamkar  2015 | RCT  Iran | PLDD | 100 | 39.7 ± 9.2 | 82/18 | 12 | NA | NA | NA | NA | 7 |
|  |  |  | OD | 100 | 40.2 ± 8.8 | 78/22 | 12 | NA | NA | NA | NA | 8 |
| 20 | Sebastian Ruetten  2008 | RCT  Germany | PELD | 91 | NA | NA | 24 | 22 ±8.25 | NA | 3 | 6 | 6 |
|  |  |  | MD | 87 | NA | NA | 24 | 43 ±9.5 | NA | 12 | 5 | 5 |
| 21 | Sebastian Ruetten  2009 | RCT  Germany | MED | 50 | 39 (23-59) | 56/44 | 24 | 24 ±7.0 | NA | 3 | NA | 5 |
|  |  |  | OD | 50 | 39 (23-60) |  | 24 | 58.0 ±13.0 | NA | 10 | NA | 5 |
| 22 | Tycho Tullberg  1993 | RCT  Sweden | MED | 30 | 40 (17-59) | 18/12 | 12 | 60.0 ±16.25 | 47±47.5 | 3 | NA | 1 |
|  |  |  | OD | 30 | 38 (18-64) | 21/9 | 12 | 46.0 ±18.75 | 47±47.5 | 1 | NA | 1 |
| 23 | Tsung-Jen Huang  2005 | RCT  China | MED | 10 | 39.2± 10.8 | 6/4 | 18.9 | 109 ±35.9 | 87.5 ±69.4 | 1 | 0 | 0 |
|  |  |  | OD | 12 | 39.8± 11.0 | 9/3 | 18.9 | 72.1±17.8 | 190 ±115 | 0 | 1 | 0 |
| 24 | Yu-Mi Ryang  2008 | RCT  Germany | MD | 30 | 39.1 ±11.3 | 19/11 | 26 | 92 ±28.6 | 63.8 ±86.8 | 2 | 4 | 4 |
|  |  |  | TD | 30 | 38.2 ±9.3 | 13/17 | 26 | 82 ±25.1 | 26.2 ±29.7 | 0 | 2 | 2 |
| 25 | Zhimin Pan  2016 | RCT  China | PELD | 48 | 39.5 (22-58) | 26/22 | 16.7 | 64.8 ±16.2 | 13.8 ±3.6 | 0 | 3 | 0 |
|  |  |  | OD | 58 | 42.8 (27-61) | 31/27 | 17.3 | 72.0 ±12.6 | 87.2 ±32.3 | 2 | 10 | 0 |
| 26 | Zihao Chen  2018 | RCT  China | PELD | 80 | 40.2 ± 11.4 | 52/28 | 12 | 97.2 ± 45.8 | NA | 8 | 3 | 5 |
|  |  |  | MED | 73 | 40.7 ± 11.1 | 37/36 | 12 | 91.7 ± 42.5 | NA | 9 | 3 | 3 |
| 27 | Zhen-mei Ding  2017 | RCT  China | PELD | 50 | 41.32 ±11.53 | 30/20 | 12 | 74.4±39.6 | NA | NA | 1 | 0 |
|  |  |  | OD | 50 | 43.90 ±11.8 | 27/23 | 12 | 66±22.8 | NA | NA | 3 | 0 |

APLD: automated percutaneous lumbar discectomy; CN: chemonucleolysis; MD: microdiscectomy; MED: microendoscopic discectomy;

OD: open discectomy; PELD percutaneous endoscopic lumbar discectomy; PLDD: percutaneous laser disc decompression; TD: tubular diskectomy.
