# Supplementary material for: Eight Surgical Interventions for Lumbar Disc Herniation: A Network Meta-Analysis on Complications
Source: Front Surg. 2021 Jul 20;8:679142. doi: 10.3389/fsurg.2021.679142 (PMC8329383; doi:10.3389/fsurg.2021.679142)
Supplement: Supplementary file 4 [file Table_4.docx]

**Node splitting analyses of Complication**

**Intra-operation**

| **Name** | **Direct Effect** | **Indirect Effect** | **Overall** | **P-Value** |
| --- | --- | --- | --- | --- |
| MD, MED | 0.67 (-0.76, 1.98) | -0.77 (-2.35, 0.90) | 0.04 (-1.17, 1.32) | 0.15 |
| MD, OD | -0.70 (-3.19, 1.74) | 0.33 (-1.15, 1.77) | 0.01 (-1.36, 1.37) | 0.41 |
| MD, PELD | -0.92 (-2.54, 0.78) | 0.10 (-2.00, 2.05) | -0.54 (-1.81, 0.75) | 0.42 |
| MED, OD | -0.02 (-1.02, 0.82) | -0.31 (-2.96, 2.52) | -0.02 (-0.90, 0.81) | 0.84 |
| MED, PELD | -0.21 (-2.15, 1.69) | -0.91 (-2.54, 0.84) | -0.59 (-1.80, 0.65) | 0.57 |
| OD, PELD | -0.38 (-2.87, 1.93) | -0.62 (-2.27, 1.13) | -0.56 (-1.86, 0.79) | 0.87 |

**Out-operation**

| **Name** | **Direct Effect** | **Indirect Effect** | **Overall** | **P-Value** |
| --- | --- | --- | --- | --- |
| MD, MED | 0.01 (-1.07, 1.16) | 0.19 (-1.14, 1.73) | 0.18 (-0.75, 1.10) | 0.77 |
| MD, OD | -0.40 (-1.91, 0.93) | 0.50 (-0.55, 1.64) | 0.11 (-0.80, 1.11) | 0.21 |
| MD, PELD | 0.58 (-0.73, 2.04) | -0.97 (-2.40, 0.47) | -0.09 (-1.04, 0.93) | 0.10 |
| MED, OD | -0.30 (-1.11, 0.49) | 1.02 (-0.65, 2.61) | -0.07 (-0.83, 0.77) | 0.13 |
| MED, PELD | -0.09 (-2.34, 2.14) | -0.35 (-1.51, 0.88) | -0.27 (-1.28, 0.80) | 0.85 |
| OD, PELD | -0.87 (-2.08, 0.28) | 0.68 (-0.72, 2.12) | -0.20 (-1.14, 0.72) | 0.09 |

**Overall complication**

| **Name** | **Direct Effect** | **Indirect Effect** | **Overall** | **P-Value** |
| --- | --- | --- | --- | --- |
| MD, MED | 0.30 (-1.05, 1.64) | 0.07 (-1.46, 1.74) | 0.25 (-0.78, 1.35) | 0.80 |
| MD, OD | -0.39 (-2.10, 1.35) | 0.67 (-0.56, 2.00) | 0.28 (-0.83, 1.42) | 0.28 |
| MD, PELD | 0.19 (-1.38, 1.94) | -0.74 (-2.48, 0.98) | -0.26 (-1.37, 0.92) | 0.41 |
| MED, OD | -0.15 (-1.10, 0.79) | 0.71 (-1.18, 2.62) | 0.02 (-0.82, 0.90) | 0.39 |
| MED, PELD | -0.21 (-2.43, 1.99) | -0.62 (-2.01, 0.80) | -0.51 (-1.62, 0.60) | 0.74 |
| OD, PELD | -1.07 (-2.58, 0.35) | 0.07 (-1.50, 1.77) | -0.53 (-1.60, 0.53) | 0.28 |

MD: microdiscectomy; MED: microendoscopic discectomy; OD: open discectomy; PELD percutaneous endoscopic lumbar discectomy.
