# Supplementary material for: Eight Surgical Interventions for Lumbar Disc Herniation: A Network Meta-Analysis on Complications
Source: Front Surg. 2021 Jul 20;8:679142. doi: 10.3389/fsurg.2021.679142 (PMC8329383; doi:10.3389/fsurg.2021.679142)
Supplement: Supplementary file 6 [file Table_6.docx]

**Node splitting analyses of reoperation**

| **Name** | **Direct Effect** | **Indirect Effect** | **Overall** | **P-Value** |
| --- | --- | --- | --- | --- |
| MD, MED | 0.04 (-1.21, 1.19) | -0.28 (-1.55, 1.04) | -0.09 (-0.96, 0.78) | 0.71 |
| MD, OD | -0.54 (-2.02, 0.60) | 0.58 (-0.51, 1.40) | 0.04 (-0.85, 0.85) | 0.13 |
| MD, PELD | 0.03 (-1.16, 1.21) | 0.26 (-1.33, 1.82) | 0.08 (-0.81, 0.99) | 0.80 |
| MD, PLDD | 1.52 (0.16, 2.80) | -0.41 (-2.16, 1.26) | 0.80 (-0.44, 1.89) | 0.08 |
| MED, OD | -0.04 (-0.94, 0.80) | 0.51 (-1.02, 1.97) | 0.12 (-0.69, 0.85) | 0.47 |
| MED, PELD | 0.71 (-1.37, 2.45) | 0.11 (-1.13, 1.26) | 0.14 (-0.83, 1.18) | 0.58 |
| OD, PELD | 0.02 (-1.62, 1.83) | 0.08 (-1.17, 1.49) | 0.04 (-0.88, 1.05) | 0.94 |
| OD, PLDD | -0.17 (-1.66, 1.36) | 1.79 (0.19, 3.45) | 0.77 (-0.42, 1.93) | 0.07 |
