# Supplementary material for: Eight Surgical Interventions for Lumbar Disc Herniation: A Network Meta-Analysis on Complications
Source: Front Surg. 2021 Jul 20;8:679142. doi: 10.3389/fsurg.2021.679142 (PMC8329383; doi:10.3389/fsurg.2021.679142)
Supplement: Supplementary file 7 [file Table_7.docx]

**Rank possibility of reoperation**

| **Intervertion** | **Rank 1** | **Rank 2** | **Rank 3** | **Rank 4** | **Rank 5** | **Rank 6** | **Rank 7** | **Rank 8** |
| --- | --- | --- | --- | --- | --- | --- | --- | --- |
| APLD | 0.24 | 0.40 | 0.13 | 0.05 | 0.03 | 0.03 | 0.03 | 0.10 |
| CN | 0.67 | 0.28 | 0.03 | 0.01 | 0.00 | 0.00 | 0.00 | 0.00 |
| MD | 0.00 | 0.01 | 0.05 | 0.15 | 0.25 | 0.24 | 0.22 | 0.09 |
| MED | 0.00 | 0.01 | 0.05 | 0.12 | 0.17 | 0.20 | 0.23 | 0.21 |
| OD | 0.00 | 0.01 | 0.07 | 0.23 | 0.21 | 0.20 | 0.18 | 0.11 |
| PELD | 0.01 | 0.03 | 0.13 | 0.20 | 0.17 | 0.16 | 0.15 | 0.15 |
| PLDD | 0.08 | 0.24 | 0.46 | 0.10 | 0.05 | 0.03 | 0.02 | 0.02 |
| Tubular Diskectomy | 0.01 | 0.02 | 0.09 | 0.15 | 0.12 | 0.14 | 0.16 | 0.32 |

APLD: automated percutaneous lumbar discectomy; CN: chemonucleolysis; MD: microdiscectomy; MED: microendoscopic discectomy; OD: open discectomy; PELD percutaneous endoscopic lumbar discectomy; PLDD: percutaneous laser disc decompression.
