# Supplementary material for: Eight Surgical Interventions for Lumbar Disc Herniation: A Network Meta-Analysis on Complications
Source: Front Surg. 2021 Jul 20;8:679142. doi: 10.3389/fsurg.2021.679142 (PMC8329383; doi:10.3389/fsurg.2021.679142)
Supplement: Supplementary file 8 [file Table_8.docx]

| **Name** | **Direct Effect** | **Indirect Effect** | **Overall** | **P-Value** |
| --- | --- | --- | --- | --- |
| MD, MED | -46.34 (-229.71, 144.18) | -171.14 (-464.63, 119.06) | -82.90 (-227.66, 71.24) | 0.40 |
| MD, OD | -58.51 (-319.80, 207.55) | 64.61 (-165.29, 298.38) | 11.41 (-147.58, 180.08) | 0.40 |
| MED, OD | 112.63 (-17.23, 246.83) | -14.58 (-348.06, 307.64) | 94.92 (-23.45, 212.45) | 0.39 |

**Node splitting analyses of blood loss**

MD: microdiscectomy; MED: microendoscopic discectomy; OD: open discectomy.
