# Supplementary material for: Eight Surgical Interventions for Lumbar Disc Herniation: A Network Meta-Analysis on Complications
Source: Front Surg. 2021 Jul 20;8:679142. doi: 10.3389/fsurg.2021.679142 (PMC8329383; doi:10.3389/fsurg.2021.679142)
Supplement: Supplementary file 10 [file Table_10.docx]

**Node splitting analyses of blood loss**

| **Name** | **Direct Effect** | **Indirect Effect** | **Overall** | **P-Value** |
| --- | --- | --- | --- | --- |
| MD, MED | -12.94 (-46.75, 20.89) | -5.47 (-44.04, 33.06) | -11.00 (-35.95, 14.05) | 0.76 |
| MD, OD | -6.22 (-46.28, 34.11) | -18.85 (-52.05, 14.76) | -16.32 (-42.26, 9.09) | 0.61 |
| MD, PELD | -14.28 (-47.59, 20.18) | -11.97 (-56.76, 33.49) | -13.21 (-39.25, 12.38) | 0.93 |
| MED, OD | -10.13 (-33.96, 13.72) | 3.77 (-42.19, 51.01) | -5.26 (-26.42, 15.34) | 0.57 |
| MED, PELD | 5.72 (-53.79, 65.08) | -5.13 (-39.28, 28.91) | -2.27 (-31.02, 26.08) | 0.74 |
| OD, PELD | 0.76 (-40.91, 41.42) | 5.21 (-33.79, 44.01) | 3.10 (-24.21, 30.36) | 0.87 |

MD: microdiscectomy; MED: microendoscopic discectomy; OD: open discectomy; PELD percutaneous endoscopic lumbar discectomy.
