# Supplementary material for: Eight Surgical Interventions for Lumbar Disc Herniation: A Network Meta-Analysis on Complications
Source: Front Surg. 2021 Jul 20;8:679142. doi: 10.3389/fsurg.2021.679142 (PMC8329383; doi:10.3389/fsurg.2021.679142)
Supplement: Supplementary file 12 [file Data_Sheet_1.docx]

**Supplementary Figure legends**

FigureS1 Risk of bias summary: review authors’ judgement about each risk of bias item for each included studies.

FigureS2 Risk of bias graph: review authors' judgement about each risk of bias item presented as percentages across all included studies.

Figure S3 Forest plots depicting the direct and indirect results of head-to-head comparisons. Odd ratio (OR) with 95% confidence intervals (CI) was used to determine the difference between direct and indirect evidence. MD: Microdiscectomy; MED: Micro-endoscopic discectomy; OD: Open discectomy; PELD: Percutaneous endoscopic lumbar discectomy.

Figure S4 Forest plots depicting the direct and indirect results of head-to-head comparisons. Forest plots depicting the direct and indirect results of head-to-head comparisons. Odd ratio (OR) with 95% confidence intervals (CI) was used to determine the difference between direct and indirect evidence. MD: Microdiscectomy; MED: Micro-endoscopic discectomy; OD: Open discectomy; PELD: Percutaneous endoscopic lumbar discectomy; PLDD: Percutaneous laser disc decompression.

Figure S5 Forest plots depicting the direct and indirect results of head-to-head comparisons. Forest plots depicting the direct and indirect results of head-to-head comparisons. Mean difference with 95% confidence intervals (CI) was used to determine the difference between direct and indirect evidence. MD: Microdiscectomy; MED: Micro-endoscopic discectomy; OD: Open discectomy; PELD: Percutaneous endoscopic lumbar discectomy.
