# Supplementary figures and images for: Eight Surgical Interventions for Lumbar Disc Herniation: A Network Meta-Analysis on Complications
Source: Front Surg. 2021 Jul 20;8:679142. doi: 10.3389/fsurg.2021.679142 (PMC8329383; doi:10.3389/fsurg.2021.679142)

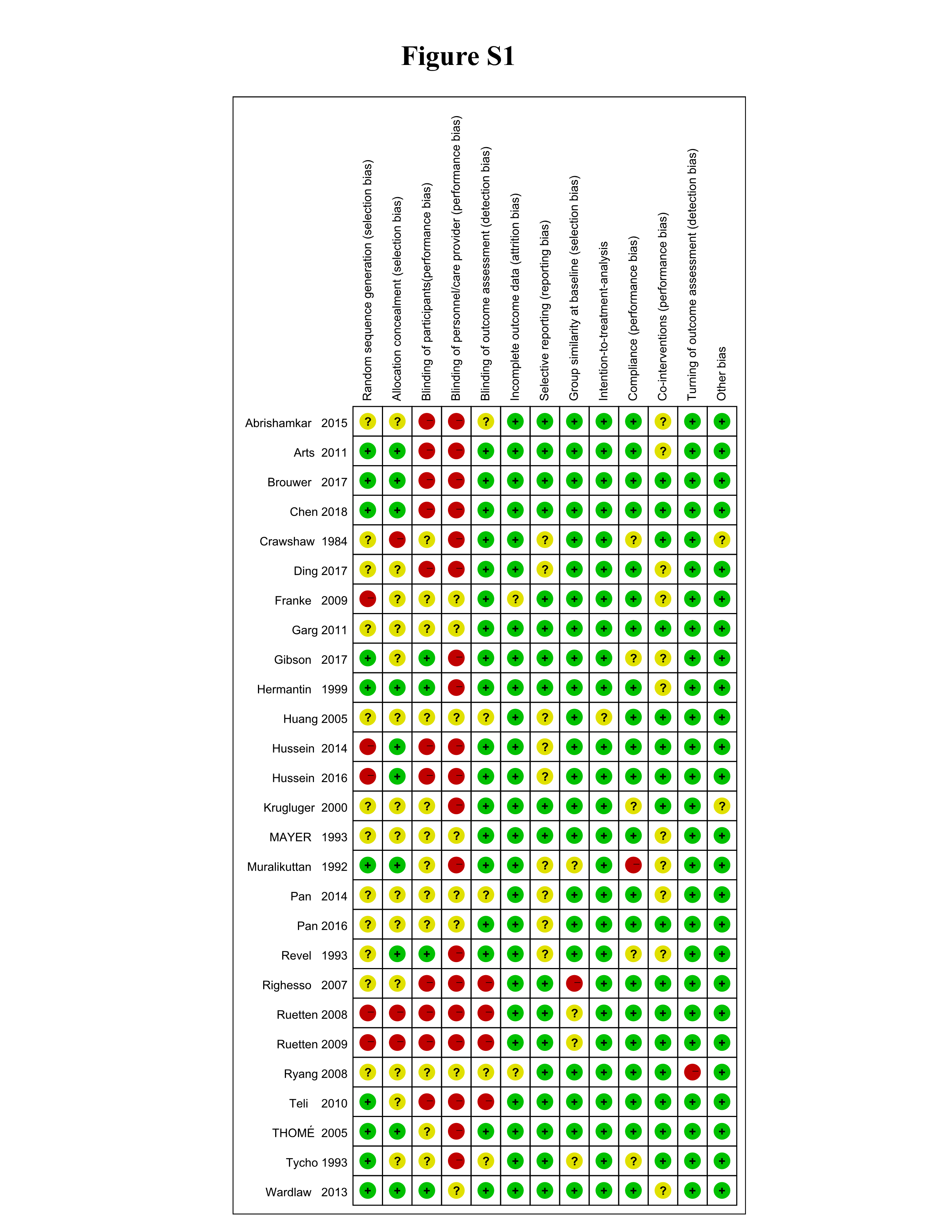

Supplement: Supplementary file 13 [file Image_1.TIF]

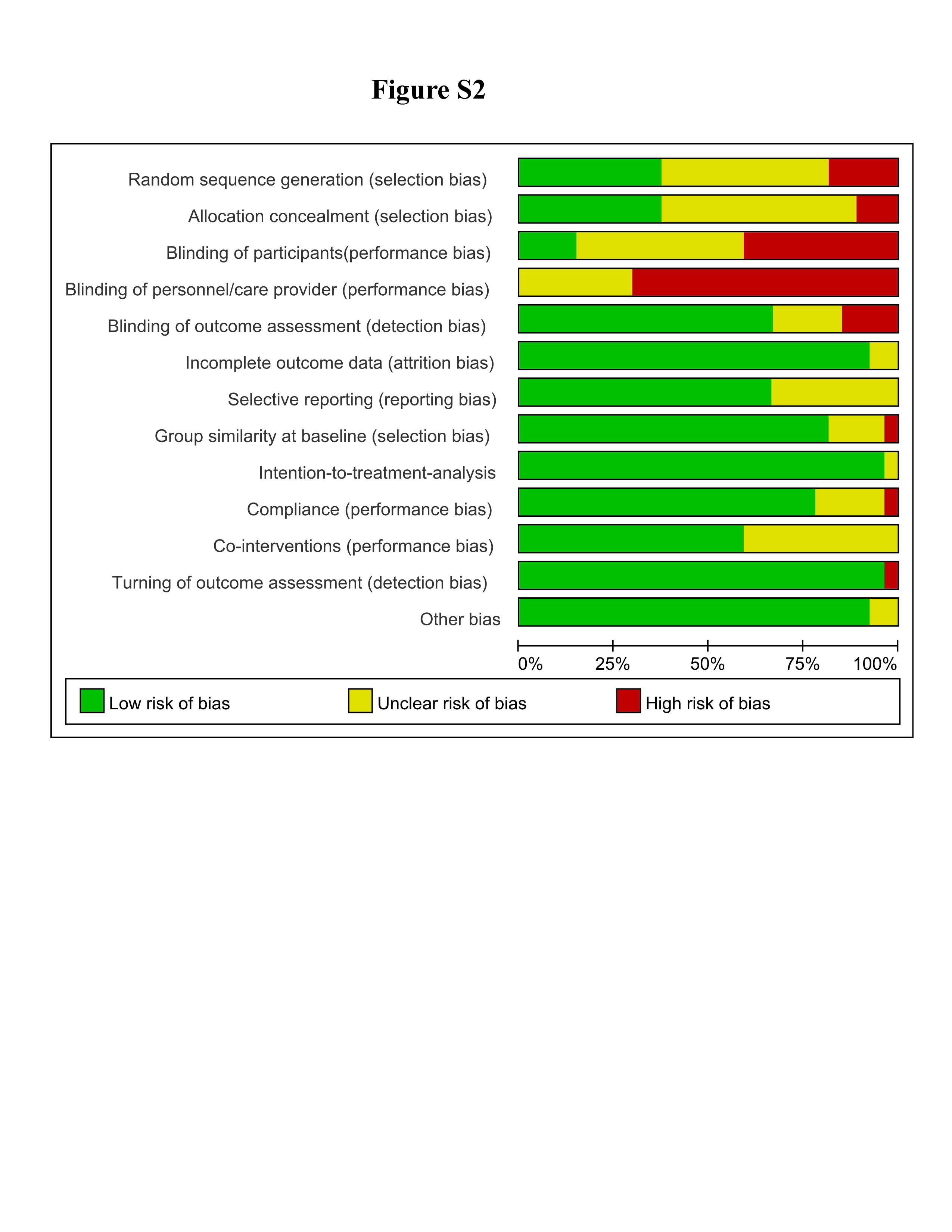

Supplement: Supplementary file 14 [file Image_2.TIF]

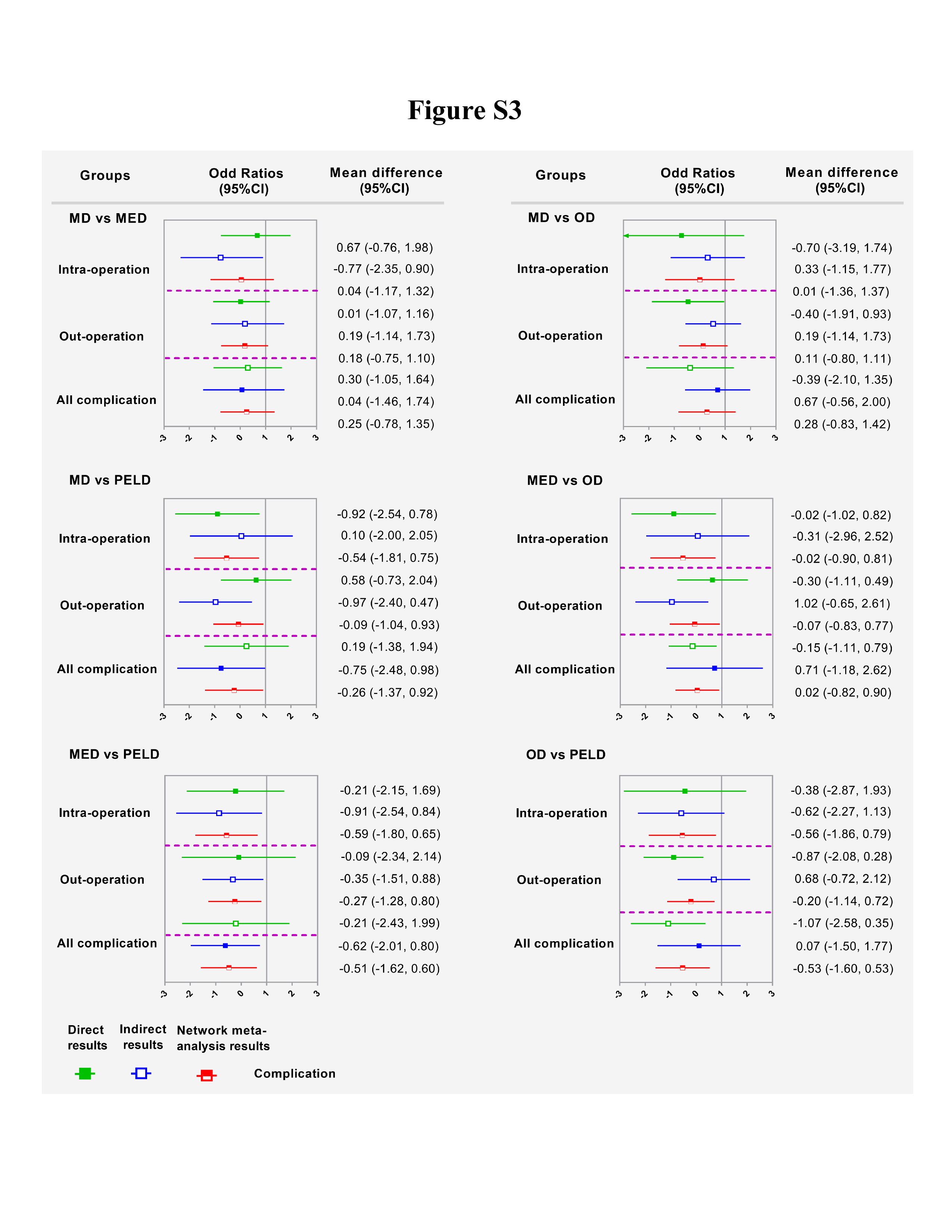

Supplement: Supplementary file 15 [file Image_3.TIF]

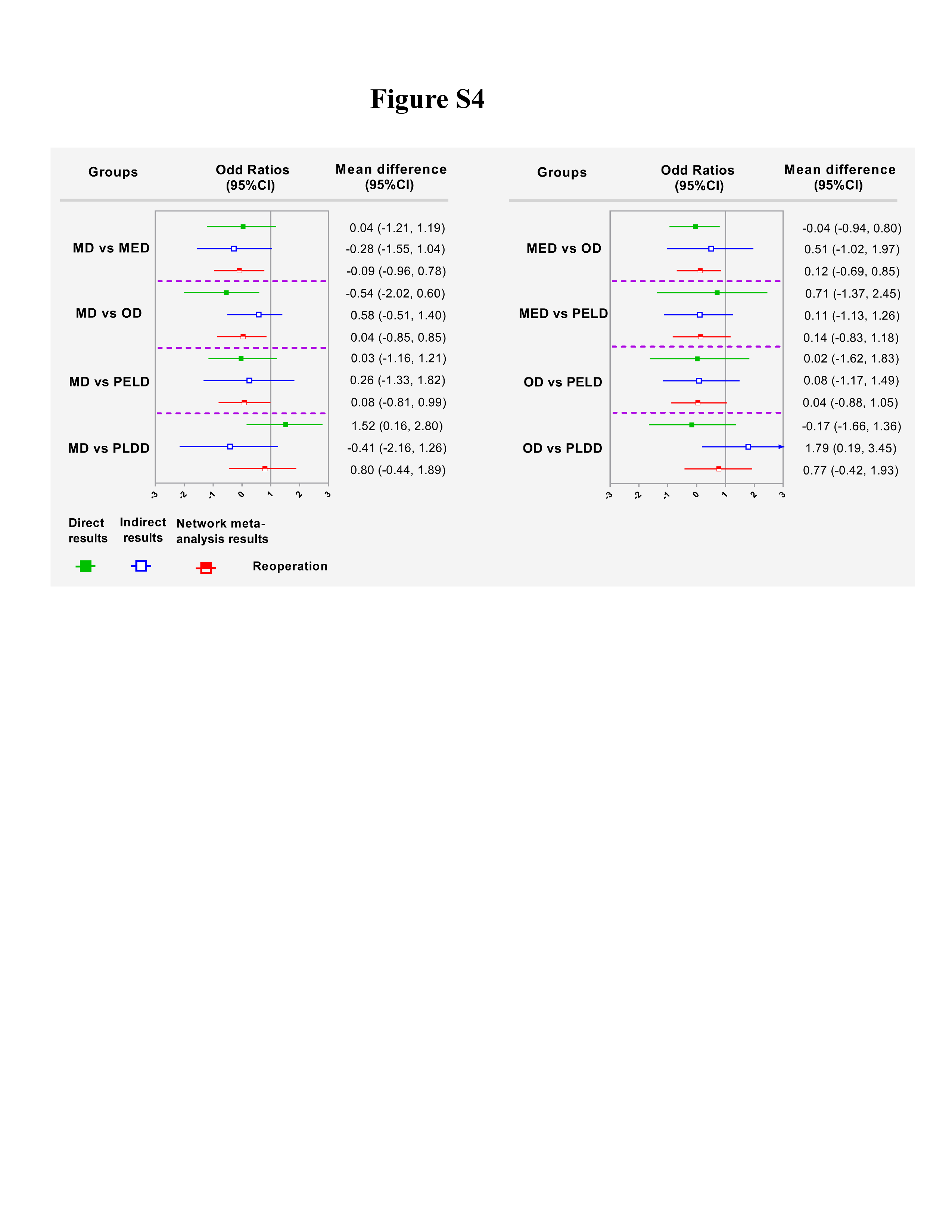

Supplement: Supplementary file 16 [file Image_4.TIF]

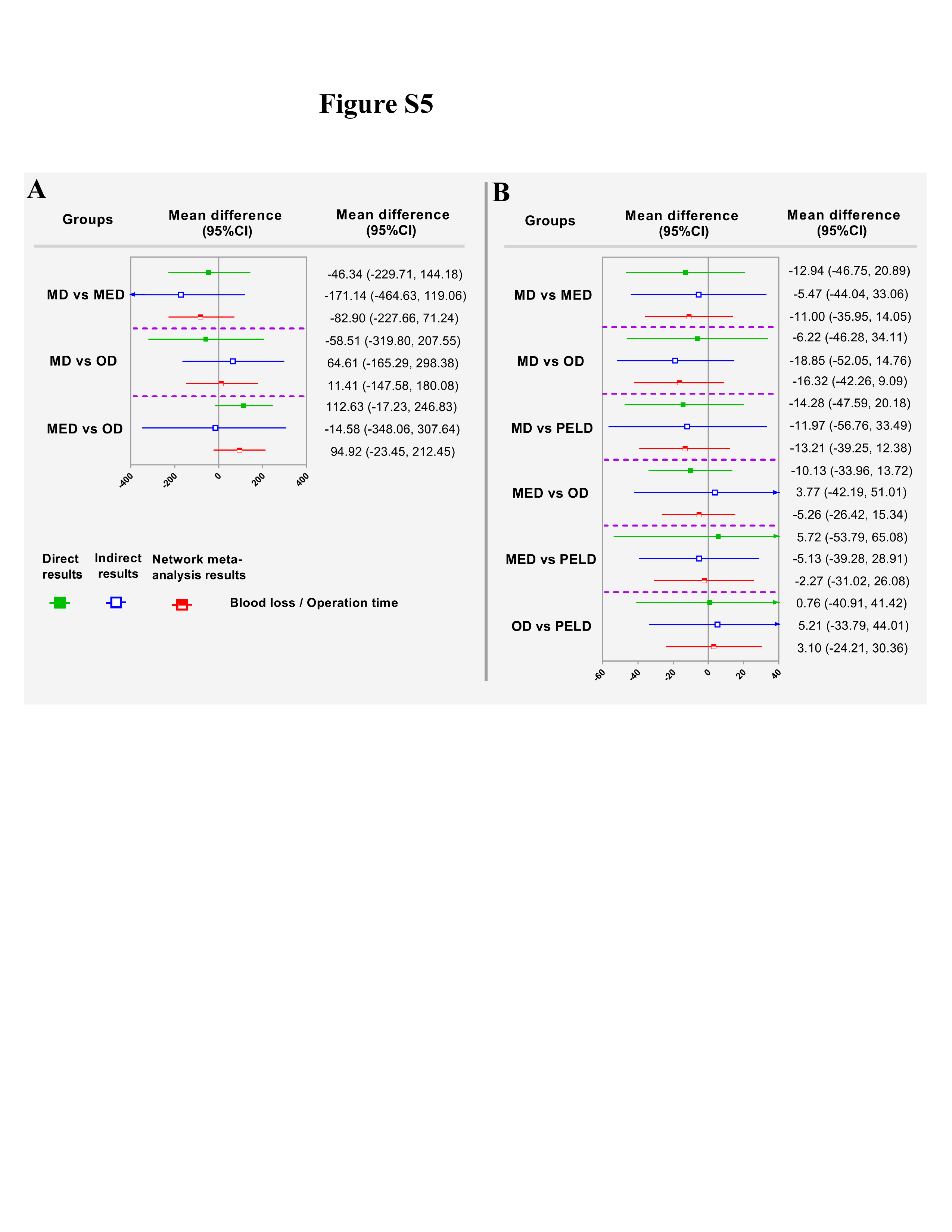

Supplement: Supplementary file 17 [file Image_5.TIF]
